# LRRK2-Mediated Neuroinflammation-Induced Neuronal Dysfunctions in a Parkinson’s and Alzheimer’s Disease Cellular Model

**DOI:** 10.3390/biom15091322

**Published:** 2025-09-16

**Authors:** Veronica Mutti, Giulia Carini, Moira Marizzoni, Alice Filippini, Federica Bono, Chiara Fiorentini, Samantha Saleri, Floriana De Cillis, Annamaria Cattaneo, Massimo Gennarelli, Paolo Martini, Isabella Russo

**Affiliations:** 1 Unit of Biology and Genetics, Department of Molecular and Translational Medicine, University of Brescia, Viale Europa 11, 25123 Brescia, Italy; veronica.mutti@unibs.it (V.M.); giulia.carini@unibs.it (G.C.); massimo.gennarelli@unibs.it (M.G.); paolo.martini@unibs.it (P.M.); 2 Biological Psychiatry Unit, IRCCS Istituto Centro San Giovanni di Dio Fatebenefratelli, 25125 Brescia, Italy; mmarizzoni@fatebenefratelli.eu (M.M.); ssaleri@fatebenefratelli.eu (S.S.); acattaneo@fatebenefratelli.eu (A.C.); 3 Genetic Unit, IRCCS Istituto Centro San Giovanni di Dio Fatebenefratelli, 25125 Brescia, Italy; afilippini@fatebenefratelli.eu; 4 Unit of Pharmacology, Department of Molecular and Translational Medicine, University of Brescia, 25123 Brescia, Italy; federica.bono@unibs.it (F.B.); chiara.fiorentini@unibs.it (C.F.); 5 Department of Pharmacological and Biomolecular Sciences, University of Milan, 20122 Milan, Italy; fdecillis@fatebenefratelli.eu

**Keywords:** neuroinflammation, LRRK2, Parkinson’s disease, Alzheimer’s disease, hiPSC, neurodegenerative diseases

## Abstract

Chronic neuroinflammation plays a crucial role in the progression of neurodegenerative diseases (NDs), including Parkinson’s disease (PD) and Alzheimer’s disease (AD). Leucine-Rich Repeat Kinase 2 (LRRK2), a gene linked to familial and sporadic PD, has been positively associated with neuroinflammation in both in vitro and in vivo systems. These observations suggest that LRRK2 might actively contribute to neuronal damage and degeneration in NDs. Based on these premises, we explored the impact of LRRK2-mediated neuroinflammation on neurons in a PD- and AD-related context. We set up a cellular model composed of human induced pluripotent stem cell (hiPSC)-derived neurons (dopaminergic for PD and cholinergic for AD) exposed to inflamed glial medium [α-synuclein pre-formed fibrils (α-syn pffs) for PD and amyloid-β (Aβ)_1–42_ fibrils for AD] for several days. To dissect the effect of neuroinflammation, and specifically, the role of LRRK2, on neuronal functions, we first performed transcriptome analysis, and then, we validated the results at functional levels. Interestingly, we found that LRRK2-dependent neuroinflammation contributes to neuronal dysfunctions and death in both ND contexts and that LRRK2 kinase inhibition prevents these detrimental effects. Overall, our results suggest that lowering neuroinflammation through LRRK2 pharmacological inhibition might limit the progression of NDs and thus be neuroprotective.

## 1. Introduction

Neuroinflammation is a cellular immune response where glial cells, microglia, and astrocytes are activated; inflammatory mediators are released; and reactive oxygen and nitrogen species are synthesized [1]. Although a fine-regulated inflammatory response represents a defense mechanism essential for brain tissue repair and integrity [2], sustained inflammation can be detrimental and lead to cell damage and degeneration [3,4]. It is well established that protracted neuroinflammation plays a crucial role in the neurodegeneration and progression of several neurodegenerative diseases (NDs), including Parkinson’s disease (PD) and Alzheimer’s disease (AD) [5]. In these pathologies, despite the different etiologies, such as infections, genetic mutations, trauma, and protein aggregations, neuronal damage is frequently associated with chronic activation of the brain’s immune response [1,6,7,8]. Indeed, chronic neuroinflammation is a prominent feature of several NDs [9].

Intriguingly, *Leucine-Rich Repeat Kinase 2* (LRRK2), a gene linked to familial and sporadic cases of PD, has been widely associated with the neuroinflammatory response [5,10]. *LRRK2* encodes for a large and complex protein containing several protein–protein domains and an enzymatic core with GTPase and kinase activities [11]. The protein is highly expressed in the brain and, particularly, in immunocompetent cells, astrocytes, and microglia compared to neuronal cells [10]. Of interest, in the last decade, numerous studies, including ours, have shown that LRRK2 kinase activity positively regulates the inflammatory response in brain immune cells in both in vitro and in vivo models upon different inflammatory challenges [10,12,13,14,15]. Specifically, it has been reported that LRRK2 kinase inhibition attenuates inflammatory response in astrocytic and microglial cells exposed to amyloid-β (Aβ)_1–42_ fibrils, α-synuclein pre-formed fibrils (α-syn pffs), LPS, manganese, and HIV-1 Tat protein [5,10,13,16,17]. Moreover, we demonstrated that two LRRK2 inhibitors, MLi2 and PF-06447475 (PF), can reduce neuroinflammation processes, including microgliosis, astrogliosis, and the level of inflammatory mediators, even in mice with intracerebral injection of α-syn pffs or Aβ_1–42_ fibrils [12]. Overall, these results indicate that LRRK2 controls the neuroinflammatory response and, importantly, suggest that LRRK2-dependent neuroinflammation might contribute to neuronal dysfunctions and degeneration observed in NDs.

Based on previous observations, in this study, we explored the effect of LRRK2-mediated neuroinflammation on neuronal functions in a context related to PD and AD by using hiPSC-derived neurons exposed to inflamed glial media as a cellular model.

In order to dissect the impact of LRRK2-mediated neuroinflammation on neurons, we implemented RNA-Seq analysis on hiPSC-derived dopaminergic (DA) neurons exposed to α-syn pff-inflamed glial medium (context related to PD) and on hiPSC-derived cholinergic (CHOL) neurons exposed to Aβ_1–42_ fibril-inflamed glial medium (context related to AD), where glial cells were activated in the presence or absence of LRRK2 kinase inhibitors MLi2 and PF. Interestingly, Gene Set Enrichment Analysis (GSEA) revealed that α-syn pff-mediated inflammation mainly activated oxidative stress- and apoptotic-related pathways in hiPSC-DA neurons, Aβ fibril-mediated inflammation induced cell shape rearrangements and apoptotic processes in hiPSC-CHOL neurons, and glial LRRK2 participates in mediating these neuronal detrimental effects. Moreover, we confirmed the effects of LRRK2-mediated neuroinflammation on neurons through validation of the RNA-Seq results on hiPSC-derived neurons at functional levels.

Overall, our findings indicate that LRRK2-dependent neuroinflammation contributes to neuronal dysfunctions and death in both NDs and, importantly, suggest that LRRK2 kinase inhibition, by attenuating neuroinflammation, might be neuroprotective.

## 2. Materials and Methods

### 2.1. Mixed Glial Primary Culture

C57BL/6J wild-type (WT) mice were maintained under a 12 h light–dark cycle at room temperature (RT, 22 °C) with ad libitum food and water. Animal procedures were performed in accordance with European Community Directive 2010/63/UE and approved by the Ethics Committee of the University of Brescia (Project ID: 800-2017, date: 23 October 2017). Moreover, the research protocol was approved by the Ethics Committee of IRCCS San Giovanni di Dio–Fatebenefratelli (n° 90-2021 and Prot. 290/2021; date: 20 October 2021).

Mixed glial primary cultures were obtained from the brains of post-natal C57BL/6J WT mice between days 1 and 4 (P1–P4). Briefly, cerebral cortices were dissociated in cold PBS, the cell suspension was maintained at room temperature (RT) for 5 min, and the top fraction was centrifuged at 1000 rpm for 5 min. The cells were then resuspended in a medium containing high-glucose DMEM (Immunological Sciences, Rome, Italy), 10% fetal bovine serum (FBS, Thermo Fisher Scientific, Waltham, MA, USA), 2mM l-Glutamine (Thermo Fisher Scientific), and penicillin–streptomycin (Thermo Fisher Scientific). Cells obtained from five brains were then seeded in 175 cm^2^ flasks and maintained at 37 °C with 5% CO_2_. After 4 days, the medium was changed, and the cells were maintained in culture until confluence (DIV8-10); then, they were processed for experimental applications. Mixed glial culture was verified by immunostaining with CD11b and glial fibrillar acidic protein (GFAP) antibodies, microglia, and astrocyte markers.

### 2.2. Aβ_1–42_ Fibril and α-Syn Pff Generation

Aβ_1–42_ fibrils were generated as previously reported [12,13]. Briefly, human Aβ_1–42_ (Bachem, Bubendorf, Switzerland) was resuspended in cold hexafluoroisopropanol (HFIP, Merck, Darmstadt, Germany; Sigma-Aldrich, St. Louis, MO, USA) and maintained under rotation at RT overnight (ON). The resulting solution was aliquoted and dried until use. Before treatment, Aβ_1–42_ was dissolved in anhydrous dimethylsulfoxide (DMSO, Merck/Sigma-Aldrich) and sonicated for 10 min at RT to remove possible peptide aggregation. Then, to obtain a fibril-enriched preparation, Aβ_1–42_ was further resuspended in 10 mM HCl and incubated at 37 °C for 48 h. α-syn pffs were generated as previously reported [12]. In detail, human monomeric α-syn (Proteos, Kalamazoo, MI, USA) was dissolved in PBS at 5 mg/mL and incubated at 37 °C for 7 days under constant shaking to induce aggregation. Enriched pffs were isolated from the soluble part of the preparation using centrifugation at 14,000 rpm for 15 min and then quantified as previously described [18]. Aβ_1–42_ fibril and α-syn pff fibrillizations were verified using the ThioflavinT assay as previously reported [12]. Before treatment, α-syn pffs were sonicated for 5 s on and 5 s off for a total of 30 s by using a 50/60 Hz ultrasonic bath (J.P. Selecta).

### 2.3. Compound and Cell Treatment

LRRK2 kinase inhibitors were dissolved in DMSO. MLi-2 and PF were used at 200 nM. Specifically, glial cells were exposed to LRRK2 kinase inhibitors for 90 min and successively treated with Aβ_1–42_ fibrils or α-syn pffs at 2 µM or 1 µM, respectively, for 16 h. To evaluate the effects of LRRK2-mediated neuroinflammation on neuronal functions, the inflamed glial medium was then taken and placed on hiPSC-derived CHOL neurons for 3 days and on hiPSC-derived DA neurons for 7 days.

### 2.4. Dopaminergic and Cholinergic Differentiation from Human iPSCs

Human iPSCs derived from a commercial certificated control (CTRL, Gibco^®^ Episomal hiPSC Line, Cat#A18945) were differentiated into DA and CHOL neurons. Briefly, hiPSC differentiation into DA neurons was performed using a modified version of the dual-SMAD inhibition protocol [19] with some modifications, as described recently [20,21,22]. After 50 days of differentiation, the mixed neuronal cultures contained ~40% neurons expressing the dopaminergic marker tyrosine hydroxylase (TH) enzyme. Specifically, for the quantification of TH-positive neurons, we examined at least one hundred neurons for each coverslip. Data are representative of at least three independent experiments performed on three different hiPSC differentiations and are expressed as the mean ± SEM.

hiPSC differentiation into CHOL neurons was based on the Krajka protocol [23] with some modifications. Briefly, hiPSCs were cultured in StemFlex medium (Thermo Fisher Scientific, Waltham, MA, USA), and when they reached 80% confluency, they were then seeded on Matrigel substrate (Corning, NY, USA). Neuronal induction (day 0) was initiated via the dual-SMAD inhibition [24] by adding SB-431542 (10 µM, Tocris, Bristol, UK; TGF-b inhibitor) and LDN-193189 (100 nM, Sigma Aldrich, St. Louis, MO, USA; BMP-inhibitor). Subsequently, to induce rostral–ventral patterning, the small molecules XAV-939 (1 µM, PeproTech, Hamburg, Germany) and purmorphamine (Pu; 1 µM PeproTech, Hamburg, Germany) were added to KSR medium [composed of KO-DMEM F12, 15% KSR, 1% P/S, 2 mM glutamine, 1% NEAA, and 0.055 mM 2-mercaptoethanol (all by Life Technologies, Carlsbad, CA, USA)]. From day 5 to 10, the KSR medium was progressively replaced every other day with the N2B27 medium [(KSR:N2B27 volume–volume ratio in percentages; day 5–6, 75:25; day 7–8, 50:50; day, 8–9 25:75; and day, 10 0:100; N2B27 medium was composed of 50% KO-DMEM F12, 50% Neurobasal, 1% B27, 1% N2 supplement, 1% P/S, 2 mM glutamine, and 1% NEAA (all by Life Technologies)]. From day 11, the cells were cultured with N2B27 medium supplemented with XAV-939 and Pu molecules. On day 20, cells were dissociated using accutase and replated on polyornithine (15 μg/mL)/laminin (1 μg/mL)/fibronectin (2 μg/mL) (all from Sigma-Aldrich) pre-coated multi-wells in N2B27 medium with brain-derived neurotrophic factor (BDNF, 20 ng/mL, PeproTech, Hamburg, Germany), glial-cell-line-derived neurotrophic factor (GDNF, 10 ng/mL; PeproTech, Hamburg, Germany), insulin-like growth factor 1 (IGF1, 10 ng/mL; PeproTech, Hamburg, Germany), cyclic adenosine monophosphate (cAMP, 500 μM; Sigma Aldrich, St. Louis, MO, USA), and Notch-pathway inhibitor (DAPT, 10 μM; Sigma Aldrich, St. Louis, MO, USA) until day 60. At the end of differentiation, ~90% of the CHOL neurons were found to express the cholinergic choline acetyltransferase enzyme (ChAT) marker. Specifically, for the quantification of ChAT-positive neurons, we examined at least one hundred neurons for each coverslip. Data are representative of at least three independent experiments performed on three different hiPSC differentiations and are expressed as the mean ± SEM.

### 2.5. RNA Extraction, Retro-Transcription, and Real-Time PCR

Total RNA from hiPSC-derived neurons was extracted using the ZymoResearch^TM^ kit (Irvine, CA, USA), according to the manufacturer’s instructions. Then, the retro-transcription reaction was performed using Moloney Murine Leukemia Virus-Reverse Transcriptase (MMLV-RT) (Thermo Fisher Scientific). Briefly, 1000 ng of total RNA was mixed with 1 µL of 0.3 µg/µL random primers (Thermo Fisher), 5 µL of 5× buffer (Thermo Fisher Scientific), 1 µL of 10 mM dNTPs (Thermo Fisher Scientific), 2.5 µL of 0.1 M DTT (Thermo Fisher Scientific), 0.5 µL of 40 U/µL RNaseOUT (Thermo Fisher Scientific), and 1 µL MMLV-RT (200 U/µL, Thermo Fisher Scientific) to a final volume of 25 µL. The reaction mixture was incubated at 37 °C for 2 h, and then, the enzyme was inactivated at 75 °C for 10 min.

To evaluate and characterize hiPSC differentiation into CHOL neurons, we analyzed the following markers: pluripotency genes NANOG and OCT4; neuronal precursors ASCL1 and DLX2; rostral–ventral forebrain progenitors FOXG1, LHX8, and NKX2; and cholinergic ChAT and VACHT. Relative gene expression levels were analyzed using GAPDH, HPRT, and ACTIN-β as housekeeping (HK) genes. The full list of primers used for real-time PCR is reported in Table 1. Real-time analyses were performed using the ViiA^TM^ 7 Real-Time PCR system (Thermo Fisher Scientific) and the iTaq Universal SYBR Green supermix (BioRad Laboratories, Hercules, CA, USA), following the manufacturer’s instructions. The relative expression of target genes was calculated using the comparative Ct (ΔΔCt) method, normalized on the geometric mean of the three HK genes, and expressed as a fold change (rq). Each individual determination was repeated in triplicate.

### 2.6. RNA Sequencing and Analysis

The quality of the RNA was assessed by Agilent 2100 Bioanalyzer (Agilent, Santa Clara, CA, USA) with the RNA 6000 Nano Kit to measure the RNA integrity number (RIN). Our samples had a RIN > 7. Libraries were prepared starting from 100 ng and by using Illumina cDNA synthesis and the Illumina Stranded mRNA Prep Ligation Kit following the manufacturer’s instructions. IDT for Illumina RNA Index Anchors and IDT for Illumina DNA/RNA UD Indexes were added to produce a dual-indexed library. The libraries were checked and quantified by using the Agilent 2100 Bioanalyzer (Agilent, Santa Clara, CA, USA) with the DNA 1000 Kit, the Qubit Fluorometer with the Qubit dsDNA HS Assay Kit, and the KAPA qPCR library quantification kit (Roche, Basel, Switzerland). Normalized and pooled libraries were sequenced on the NextSeq 1000/2000 Illumina platform using P3 Reagent Cartridge (300 Cycles). Data quality was assessed with FastQC, and transcript quantification was performed using Salmon (v1.9.0) [25] with transcripts defined in Ensembl 106. Differential gene expression levels were estimated with the comparisons: α-syn vs. CTRL, α-syn + MLI2 vs. CTRL, and α-syn + PF vs. CTRL for DA neurons; Aβ vs. CTRL, Aβ + MLi2 vs. CTRL, Aβ + PF vs. CTRL for CHOL neurons using the DESeq2 R package, including RUV weights as factors in the models [26]. Transcripts with a |fold-change (FC)| > 0.5 and q-values < 0.05 were classified as differentially expressed (DEGs).

Summary statistics from DESeq2 for each comparison were used to perform GSEA (gseGO function from clusterProfiler R package) to find enriched gene sets or pathways. Pathways with Normalized Enrichment Scores (NESs) ≥ 0 and q-values < 0.05 were considered significant. All analyses were performed using R v4.3.2. All plots were performed using ggplot2 v3.4.2 [27].

### 2.7. Immunofluorescence

hiPSC-derived neurons were fixed in 4% PFA for 15 min, followed by PBS washes. Cells were then blocked in PBS containing 0.1% Triton x-100 (Sigma Aldrich) and 5% bovine serum albumin (BSA; Sigma Aldrich) at RT for 1 h with the following primary antibodies: MAP2 (1:800, Immunological Science), ChAT (1:300, Sigma Aldrich), and TH (1:700, SantaCruz, Dallas, TX, USA). The day after, cells were incubated for 30 min at RT with appropriate Alexa Fluor 488- and 594-conjugated secondary antibodies (1:1000, Invitrogen, Waltham, MA, USA). Nuclei were stained with DAPI. Images were acquired by a Zeiss LSM 900 confocal microscope using a Zeiss 63×/1.4 numerical aperture oil-immersion objective (Carl Zeiss AG, Oberkochen, Germany).

### 2.8. Propidium Iodide Staining

To evaluate the effect of LRRK2-mediated neuroinflammation on hiPSC-derived DA or CHOL neuronal death, we used propidium iodide (PI) staining (Sigma Aldrich). Specifically, hiPSC-derived neurons were treated with PI (5 μg/μL) in neuronal medium at 37 °C for 15 min and then fixed with 4% PFA and subjected to TH or ChAT staining. PI-positive neurons were counted using a Zeiss Axioplan2 fluorescence microscope with a 20× objective (Carl Zeiss AG). At least one hundred TH-positive or ChAT-positive neurons were randomly analyzed in six independent experiments. Statistical significance of differences between groups was assessed using one-way ANOVA followed by Tukey’s post hoc test.

### 2.9. Reactive Oxygen Species (ROS) Detection

ROS generation was measured using the CellROX Green Reagent (Marín 2006, [11]) (Thermo Fisher Scientific), a cell-permeable fluorescent probe that detects oxidative stress in live cells, following the manufacturer’s instructions. Specifically, hiPSC-derived neurons were seeded on 96 multi-wells (1.5 × 10^4^ cells/well) or 24 multi-wells (1 × 10^4^ cells/well). At the end of differentiation, neurons were washed once with 1× PBS and treated with 5 µM CellROX Green Reagent in neuronal medium for 30 min at 37 °C. Successively, neurons were washed with 1× PBS, and the ROS fluorescence levels were detected using a PerkinElmer EnSight-Multimode Plate Reader, setting the excitation at 485 nm and the emission at 520 nm. For the immunofluorescence staining, neurons were then fixed with 4% PFA and subjected to TH staining.

### 2.10. Immunocytochemistry and Neuronal Morphometric Measurement

hiPSC-derived DA and CHOL neurons were fixed in 4% PFA for 15 min, blocked in PBS containing 0.1%Triton x-100 (Sigma Aldrich) and 5% bovine serum albumin (BSA; Sigma Aldrich), and then incubated ON at 4 °C with anti-TH (1:700) or anti-ChAT (1:300) antibodies, respectively. The day after, cells were exposed to a biotinylated anti-mouse antibody for 30 min at RT, followed by an incubation with avidin–biotin horseradish peroxidase complex (VECTOR Laboratories, Newark, CA, USA) and finally treated with diaminobenzidine (DAB Kit, VECTOR Laboratories). Digital images were acquired with the Olympus IX51 microscope and analyzed as previously described [20]. Morphometric measurements were performed on digitized images using the NIH ImageJ software 1.53. The maximal dendrite length and the soma area were analyzed as recently described [20]. Three coverslips per treatment were examined to obtain measurements from at least thirty TH-positive hiPSC-derived DA neurons or ChAT-positive hiPSC-derived CHOL neurons.

### 2.11. Cell Lysis and Western Blotting

Glial cells were washed twice with 1× PBS, solubilized with cold lysis buffer (20 mM Tris–HCl pH 7.5, 150 mM NaCl, 1 mM EDTA, 2.5 mM sodium pyrophosphate, 1 mM β-glycerophosphate, 1 mM Na_3_VO_4_, 1% Triton-X-100, and protease inhibitors), incubated on ice for 20 min, and centrifuged at 14,000 rpm at 4 °C. The supernatant was collected for protein electrophoresis. Specifically, total proteins were separated using 7.5% acrylamide Sodium Dodecyl Sulphate (SDS)-PAGE gels. Subsequently, proteins were transferred to a polyvinylidene difluoride (PVDF) membrane (Bio-Rad), saturated with 5% non-fat dry milk in 0.1% TBS-Tween (TBST) for 1 h at RT, and incubated with primary antibodies: goat anti-IL1β (R&D System, Minneapolis, MN, USA, AF-401-NA, 1:500), rabbit anti-IL6 (Thermo Fisher, 1:500), rabbit phospho serine 935 LRRK2 (Abcam, Cambridge, UK, Ab133450, 1:300), rabbit LRRK2 total (Abcam, Ab133474, 1:300), mouse anti-GAPDH (Thermo Fisher Scientific MA5-15738, 1:30,000), and mouse a-tubulin (α-tub; Abcam, 1:10,000). Next, membranes were incubated with horseradish peroxidase (HRP)-conjugated secondary antibodies (Merck-Sigma Aldrich) for 1h at RT and then with ECL substrate of HRP (GE Healthcare, Boston, MA, USA).

### 2.12. Statistical Analysis

All data are expressed as mean ± SEM and represent at least three sets of experiments. Statistical significance of differences between groups was assessed using one-way ANOVA followed by Tukey’s post hoc test. Data were analyzed using the Prism software (v8.0; GraphPad Software Inc., San Diego, CA, USA), and statistical significance was taken at *p* < 0.05.

## 3. Results

### 3.1. Generation and Characterization of hiPSC-Derived Neurons

**Figure 1:**
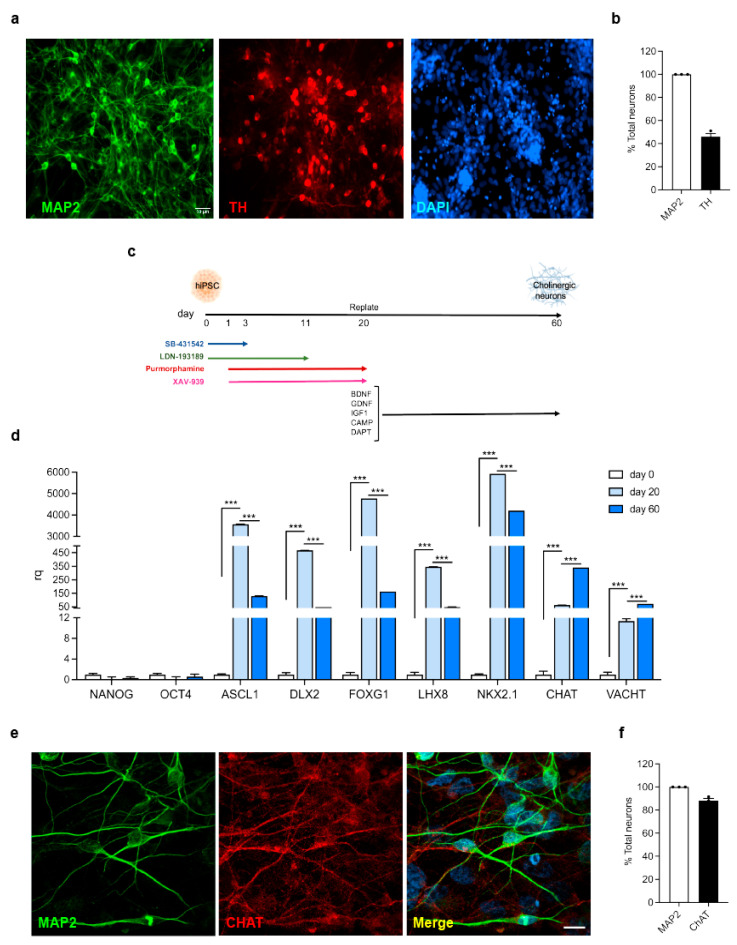
Generation and characterization of hiPSC-derived neurons. (**a**) Confocal images of hiPSC-derived DA neurons (day 50) stained for MAP2 (green), TH (red), and DAPI for nuclei (blue). Scale bar, 50 µm. (**b**) Quantification of TH-positive neurons after 50 days of differentiation. At least one hundred neurons for each coverslip were examined. Data are representative of at least three independent experiments performed on three different hiPSC differentiations and are expressed as the mean ± SEM. (**c**) Schematic representation of hiPSC differentiation into CHOL neurons. (**d**) Relative expression of NANOG, OCT4, ASCL1, DLX2, FOXG1, LHX8, NKX2, ChAT, and VACHT markers in undifferentiated hiPSC (day 0) and after 20 (day 20) and 60 days (day 60) of differentiation. Gene expression was calculated using the comparative Ct (ΔΔCt) method, normalized on the geometric mean of the three HK genes, and expressed as a fold change (rq). Data are representative of three independent experiments performed on two different hiPSC differentiations and are expressed as the mean ± SEM. Data were analyzed using one-way ANOVA followed by Tukey’s post hoc test, *** *p* < 0.0001. (**e**) Confocal images of hiPSC-derived CHOL neurons (day 70) stained for MAP2 (green), ChAT (red), and DAPI for nuclei (blue). Scale bar, 50 µm. (**f**) Quantification of ChAT-positive neurons after 70 days of differentiation. At least one hundred neurons for each coverslip were examined. Data are representative of at least three independent experiments performed on three different hiPSC differentiations and are expressed as the mean ± SEM.

To explore the effect of LRRK2-mediated neuroinflammation on neuronal functions, we set up a cellular model composed of hiPSC-derived neurons exposed to inflamed glial media. In detail, for the PD-related context, we generated and exposed hiPSC-derived DA neurons to α-syn pff-inflamed glial media, while for the AD-related context, we generated and exposed hiPSC-derived CHOL neurons to Aβ fibril-inflamed glial media. To this end, we differentiated hiPSCs into DA neurons, as widely described by our collaborators [20,21,22]. Specifically, after 50 days of differentiation, the mixed neuronal cultures contained ~40% neurons expressing the dopaminergic marker TH enzyme (Figure 1a,b). Conversely, hiPSC differentiation into CHOL neurons was achieved using a modified version of the feeder-free 2D monolayer culture system, as previously published [23] (Figure 1c). To assess the efficiency of our protocol, we evaluated gene expression for markers of pluripotency, neuronal progenitors, ventral anterior forebrain pattern, and CHOL neurons during the differentiation process using RT-PCR. As shown in Figure 1d, on day 0, undifferentiated hiPSCs expressed the pluripotency genes NANOG and OCT4. Gene expression of the neuronal progenitor markers ASCL1 and DLX2 was robustly induced on day 20, together with the ventral anterior forebrain FOXG1, LHX8, and NKX2.1 patterns. CHOL neuron-specific genes, such as ChAT and VACHT, started to be expressed on day 20 and greatly increased on day 60, indicative of neuronal differentiation and maturation (Figure 1d). We also confirmed the expression of neuronal and CHOL markers through imaging. Upon maturation (day 60), most of the cells were positive for neuronal MAP2 and for the cholinergic ChAT marker (~90% of the cells; Figure 1e,f). Overall, these results indicate the good efficiency of CHOL neuronal differentiation and maturation from hiPSCs.

### 3.2. Transcriptomic Profiles of hiPSC-Derived Neurons Exposed to Inflamed Glial Media

To explore the effect of LRRK2-mediated neuroinflammation on neuronal functions, we first set up the experimental conditions for our cellular model. Thus, we confirmed glial inflammation on α-syn pff or Aβ fibril challenge and observed a reduction in the inflammatory response after treatment with LRRK2 kinase inhibitors MLi2 and PF. Specifically, mixed glial cells were pre-treated with LRRK2 inhibitors (MLi2 or PF) at 200 nM for 90 min and, subsequently, activated with 1 μM α-syn pffs or 2 μM Aβ fibrils for 16 h. As clearly shown in our last published papers [12,13,15], we validated that LRRK2 MLi2 or PF inhibitor can reduce glial inflammatory mediators (IL-1β, IL-6, and ROS) on α-syn pffs and Aβ fibrils (Supplementary Figure S1). Moreover, we confirmed the inhibition of LRRK2 kinase activity after MLi2 or PF treatment in glial cells by assessing LRRK2 phospho serine 935 levels (Supplementary Figure S2). Then, we performed a time-course study with hiPSC-derived neurons exposed to inflamed glial medium to identify the time of treatment to use in the following experiments (Supplementary Figure S3a). To this end, mature hiPSC-derived DA and CHOL neurons were exposed to α-syn pff- and Aβ fibril-inflamed glial medium, respectively, for 1, 3, and 7 days, and we evaluated neuronal ROS generation by using CellROX Green. We found that α-syn inflammation triggered a robust induction of ROS production starting at day 1, which continued to increase on day 7 of treatment compared to untreated hiPSC-derived DA neurons (Supplementary Figure S3b). Aβ inflammation induced an increase in ROS production from day 1 to day 3 of treatment compared to the untreated hiPSC-derived CHOL neurons, which then remained stable on day 7 (Supplementary Figure S3c). Based on these observations, to explore the effects of LRRK2-mediated neuroinflammation on neuronal functions, we decided to expose hiPSC-derived DA neurons to α-syn pff-inflamed glial medium for 7 days and hiPSC-derived CHOL neurons to Aβ fibril-inflamed glial medium for 3 days.

**Figure 2:**
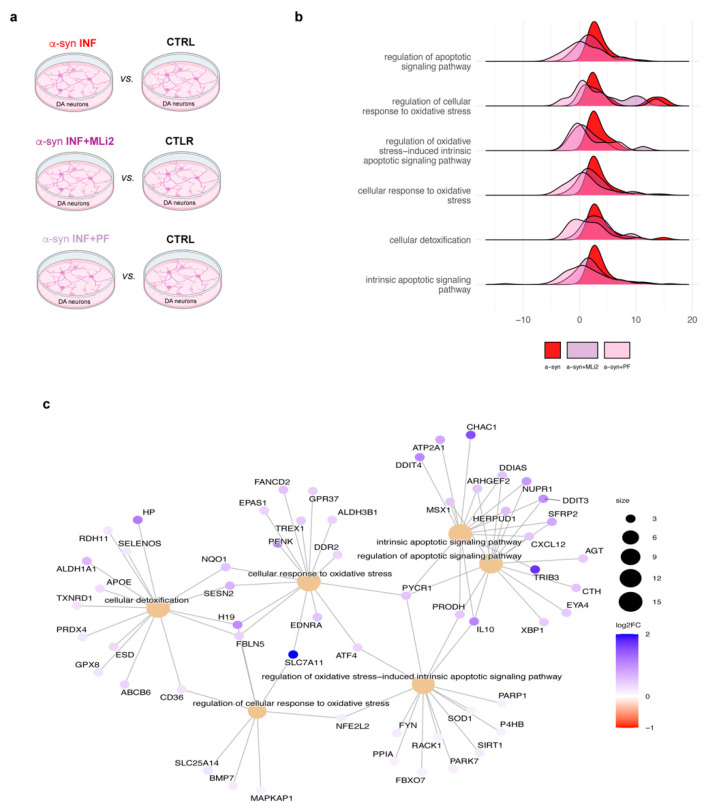
Overview of the α-syn pff-related RNA Seq data analysis. (**a**) RNA Seq analysis comparisons. (**b**) Ridge plots for selected GO biological processes in neurons exposed to α-syn pff inflammation. Core enrichment genes from neurons exposed to α-syn pff inflammation vs. neurons exposed to control medium comparison are plotted using the statistics distribution of α-syn vs. CTRL in red overlay, with α-syn + MLi2 vs. CTRL in violet and α-syn + PF vs. CTRL in pink. (**c**) Cnet-plot showing the top 15 genes with greater log2 fold changes in the selected pathways for neurons exposed to α-syn pff inflammation.

Then, to dissect the effects of LRRK2-mediated neuroinflammation on neuronal functions, we explored transcriptome profiles of hiPSC-derived neurons exposed to inflamed medium derived from glial cells activated in the presence or absence of LRRK2 inhibitors. We started from hiPSC-derived DA neurons exposed to α-syn pff inflammation for 7 days. By comparing hiPSC-derived DA neurons exposed to α-syn inflammation vs. neurons exposed to control medium (α-syn vs. CTRL), we found 635 DEGs (259 up- and 376 down-regulated). When we compared hiPSC-derived DA neurons exposed to α-syn inflammation in the presence of MLi2 inhibitor vs. neurons exposed to control medium (α-syn + MLi2 vs. CTRL), we observed 1413 DEGs (760 up- and 653 down-regulated) and, similarly, 1628 DEGs (983 up- and 645 down-regulated) when we compared hiPSC-derived DA neurons exposed to α-syn inflammation in the presence of PF inhibitor vs. neurons exposed to control medium (α-syn + PF vs. CTRL; Figure 2a; Supplementary Table S1). Of interest, we found that many DEGs were specifically activated by α-syn inflammation. In detail, we observed 64 up-regulated DEGs that were activated by α-syn inflammation vs. neurons exposed to control medium but not significantly up-regulated in the presence of LRRK2 inhibitors. This number increases to 181 up-regulated DEGs if we consider DEGs not up-regulated in at least one condition with inhibitors, either MLi2 or PF. Intriguingly, among the up-regulated DEGs not activated in the presence of LRRK2 inhibitors, we observed genes related to oxidative stress, like EDNRA, and apoptosis, like ARHGEF2.

**Figure 3:**
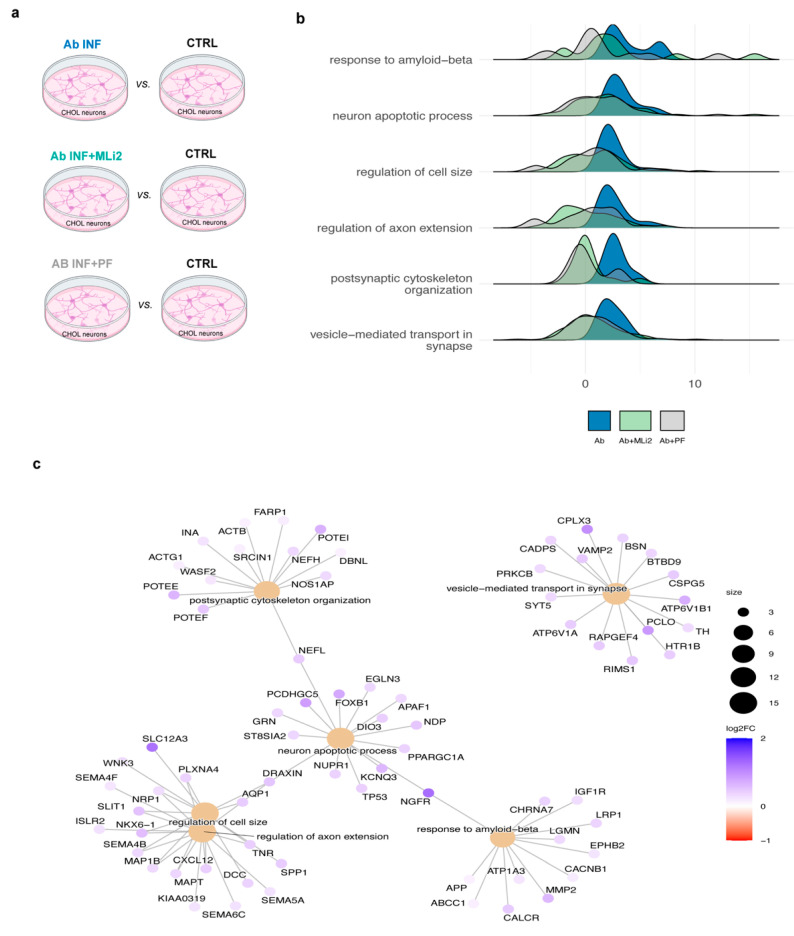
Overview of the Aβ-related data analysis. (**a**) RNA Seq analysis comparisons. (**b**) Ridge plots for selected GO biological processes in neurons exposed to Aβ inflammation. Core enrichment genes from Aβ vs. CTRL comparison are plotted using the statistics distribution of Aβ vs. CTRL in blue overlay with Aβ + MLi2 vs. CTRL in light green and Aβ + PF vs. CTRL in gray. (**c**) Cnet-plot showing the top 15 genes with greater log2 fold changes in the selected pathways.

To gain insight into the neuronal–biological processes affected by α-syn inflammation but protected by LRRK2 inhibition, we performed a Gene Set Enrichment Analysis (GSEA) on Gene Ontology biological processes. By comparing α-syn vs. CTRL, the GSEA analysis identified 420 pathways. In the α-syn + MLi2 vs. CTRL and α-syn + PF vs. CTRL comparisons, we found only 216 and 364 pathways (Supplementary Table S2). Interestingly, in hiPSC-derived DA neurons exposed to α-syn inflammation for 7 days, we identified cellular processes that were not activated when DA neurons were exposed to inflammation with inhibited LRRK2, including oxidative stress-related pathways, processes related to the cellular response toward detoxification, and apoptotic signaling pathways (Figure 3b). Looking at the genes that drove the differences in pathway activation, we identified, among others, some specifically up-regulated DEGs caused by α-syn inflammation, including IL-10, SFRP2, and ARHGEF2 (the others were PYCR1 and HERPUD1), related to apoptosis, and EDNRA, FANCD2 PYCR1, SESN2, H19, ALDH1A1, and HP, related to oxidative stress (Figure 3c, Supplementary Figure S4a,b). Taken together, these results suggest that LRRK2-mediated α-syn inflammation induces oxidative stress and cell degeneration in hiPSC-derived DA neurons, which could be mitigated by LRRK2 kinase inhibition.

**Figure 4:**
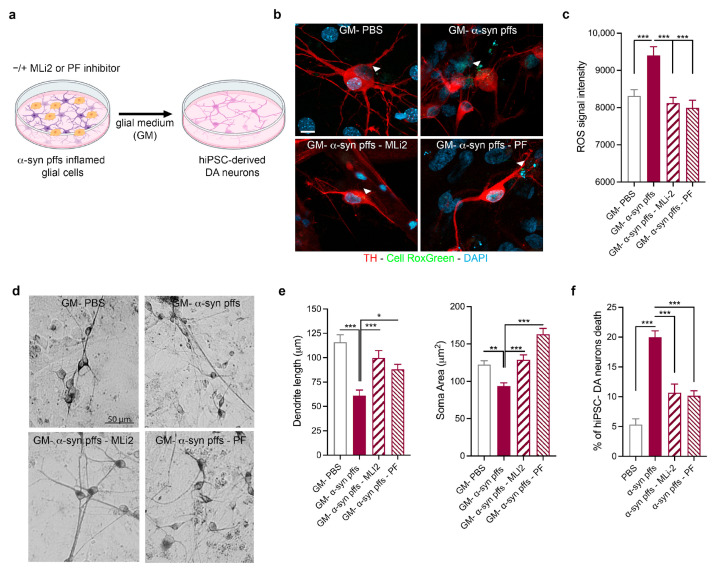
Validation of the effect of LRRK2-mediated α-syn pff neuroinflammation on hiPSC-derived DA neurons. (**a**) Schematic representation of the experimental design. (**b**) Confocal images of hiPSC-derived DA neurons exposed to glial medium (GM)–PBS; GM-α-syn pffs; GM-α-syn pffs and MLi2; and GM-α-syn pffs and PF. The white arrowhead corresponds to the ROS. Cells were treated with CellRox Green for the last 30 min of life and stained for TH (red) and DAPI for nuclei (blue). Scale bar, 50 µm. (**c**) Quantification of ROS levels in live hiPSC-derived DA neurons exposed to GM-PBS; GM-α-syn pffs; GM-α-syn pffs and MLi2; and GM-α-syn pffs and PF. Data are representative of ten independent experiments and are expressed as the mean ± SEM. Data were analyzed using one-way ANOVA followed by Tukey’s post hoc test, *** *p* < 0.0001. (**d**) Representative microphotographs of TH-positive neurons (hiPSC-derived DA neurons) following exposure to GM-PBS; GM-α-syn pffs; GM-α-syn pffs and MLi2; and GM-α-syn pffs and PF. Scale bar, 50 μm. (**e**) Morphologic effects of inflamed glial medium on dendrite length and soma area of hiPSC-derived DA neurons. Three coverslips per treatment were examined to obtain measurements from at least thirty TH-positive hiPSC-derived DA neurons. Data are expressed as the mean ± SEM and were analyzed using one-way ANOVA followed by Tukey’s post hoc test, * *p* < 0.05, ** *p* < 0.001, and *** *p* < 0.0001. (**f**) Quantification of TH-positive neuron death following exposure to GM-PBS; GM-α-syn pffs; GM-α-syn pffs and MLi2; and GM-α-syn pffs and PF. At least one hundred TH-positive neurons were randomly analyzed for PI. Data are representative of six independent experiments and are expressed as the mean ± SEM. Data were analyzed using one-way ANOVA followed by Tukey’s post hoc test, *** *p* < 0.0001.

Similarly, we analyzed hiPSC-derived CHOL neurons exposed to Aβ fibril inflammation for 3 days in the presence or absence of LRRK2 inhibitors (referred to as Aβ, Aβ + MLi2, Aβ + PF; Figure 4a). Using control medium as a reference, we found 255 DEGs (129 up- and 126 down-regulated) when we compared hiPSC-derived CHOL neurons exposed to Aβ inflammation vs. CTRL (Aβ vs. CTRL), 410 DEGs (158 up- and 252 down-regulated) in the presence of MLi2 inhibitor (Aβ + MLi2 vs. CTRL), and 956 DEGs (323 up- and 633 down-regulated) in the presence of PF inhibitor (Aβ + PF vs. CTRL; Figure 3a, Supplementary Table S3). We observed that LRRK2-inhibited Aβ inflammation prevents the up-regulation of several key genes for CHOL neurons as well. Specifically, 69 up-regulated DEGs were inhibited by both the compounds and 101 by at least one (either MLi2 or PF). Among the inhibited genes, we observed that MMP2 is involved in the response to Ab, PCLO is implicated in synaptic vesicle trafficking, and KCNQ3 encodes a potassium channel protein that is important for neuronal transmission.

With GSEA, we identified 570 biological processes comparing hiPSC-derived CHOL neurons exposed to Aβ inflammation vs. neurons exposed to control medium. In the presence of MLi2 and PF inhibitors, we observed a reduction in the significant pathways to 96 and 143 biological processes, respectively (Supplementary Table S4). As for α-syn inflammation, we observed that LRRK2-inhibited inflammation has a protective effect by preventing the activation of some relevant pathways. In detail, we observed several GO categories that were activated in hiPSC-derived CHOL neurons exposed to Aβ inflammation but protected in the presence of LRRK2 inhibitors, such as cell shape rearrangement, including cell size and axon extension, and apoptosis (Figure 3b). Then, we identified the genes that drove relevant differences in pathway activation, with SLC12A3 and SLIT1 related to cell size regulation; KCNQ3 and PCDHGC5 involved in the control of neuron apoptotic process; MMP2, CACNB1, and IGFR1 for the response to Aβ; and ATP6V1B1 and PCLO involved in vesicle-mediated transport in the synapse (Figure 3c, Supplementary Figure S5a,b). Taken together, these findings suggest that LRRK2-mediated Aβ inflammation induces neuronal shape rearrangements and degeneration in hiPSC-derived CHOL neurons.

### 3.3. Validation of the Effect of LRRK2-Mediated α-Syn Pff Neuroinflammation on hiPSC-Derived DA Neurons

We then validated the effects of LRRK2-mediated α-syn pff neuroinflammation on hiPSC-derived DA neurons. We exposed hiPSC-derived DA neurons for 7 days to α-syn pff inflammation with or without LRRK2 inhibition (MLi2 or PF; Figure 4a), and we first examined neuronal oxidative stress. As shown by the CellRox Green assay (Figure 4b,c), we found that α-syn pff inflammation induced neuronal ROS generation that was significantly reduced when glial cells were activated in the presence of LRRK2 MLi2 or PF inhibitor. These results confirm that α-syn pff neuroinflammation triggers oxidative stress in hiPSC-derived DA neurons and that glial LRRK2 takes part in this neuronal effect.

Successively, we examined the impact of LRRK2-mediated neuroinflammation on neuronal degeneration (Figure 4d–f). Thus, we measured dendrite length and soma area to evaluate a neuronal suffering phenotype. Specifically, at the end of differentiation, hiPSC-derived DA neurons were immunolabeled with an anti-TH antibody to specifically identify and measure TH neuronal morphology. As shown in Figure 4, α-syn pff inflammation leads to a significant reduction in the length of primary dendrites and the soma area of TH-positive DA neurons, the effects of which were not induced when glial cells were activated in the presence of LRRK2 MLi2 or PF inhibitor (Figure 4d,e). Interestingly, when we analyzed DA neuronal degeneration, through PI staining and TH immunolabeling, we found that α-syn pff inflammation leads to neuronal death and that, of relevance, LRRK2-inhibited inflammation (with both PF and MLi2) protects neuronal vitality (Figure 4f). Altogether, these results confirmed the RNA-Seq data and suggest that LRRK2-mediated neuroinflammation contributes to DA neuronal dysfunctions and degeneration in PD.

### 3.4. Validation of the Effect of LRRK2-Mediated Aβ Fibril Neuroinflammation on hiPSC-Derived CHOL Neurons

**Figure 5:**
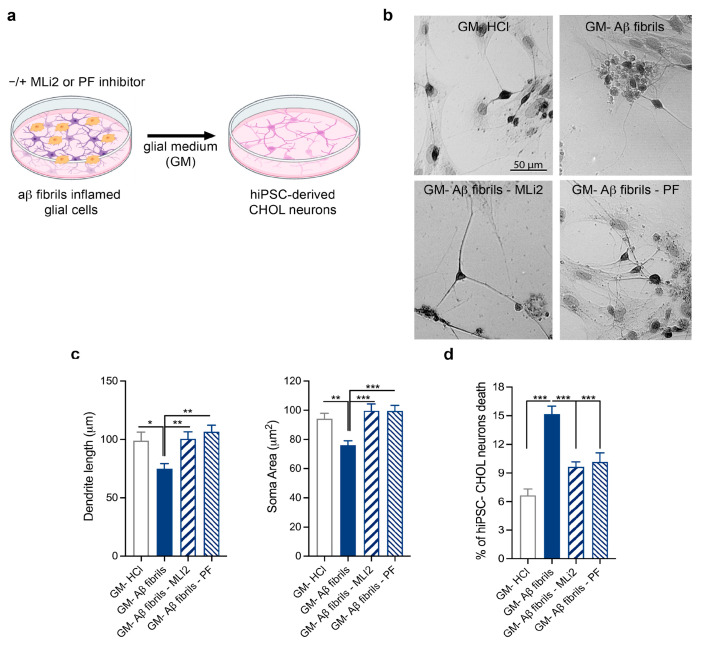
Validation of the effect of LRRK2-mediated Aβ fibril neuroinflammation on hiPSC-derived CHOL neurons. (**a**) Schematic representation of the experimental design. (**b**) Representative microphotographs of ChAT-positive neurons following exposure to GM-HCl; GM-Aβ fibrils; GM-Aβ fibrils and MLi2; and GM-Aβ fibrils and PF. Scale bar, 50 μm. (**c**) Morphologic effects of inflamed glial medium on dendrite length and soma area of hiPSC-derived CHOL neurons. Three coverslips per treatment were examined to obtain measurements from at least thirty ChAT-positive hiPSC-derived CHOL neurons. Data are expressed as the mean ± SEM and were analyzed using one-way ANOVA followed by Tukey’s post hoc test, * *p* < 0.05, ** *p* < 0.001, and *** *p* < 0.0001. (**d**) Quantification of ChAT-positive neuron death following exposure to GM-HCl; GM-Aβ fibrils; GM-Aβ fibrils and MLi2; and GM-Aβ fibrils and PF. At least one hundred ChAT-positive neurons were randomly analyzed for PI. Data are representative of six independent experiments and are expressed as the mean ± SEM. Data were analyzed using one-way ANOVA followed by Tukey’s post hoc test, *** *p* < 0.0001.

We then functionally validated the effects of LRRK2-mediated Aβ fibril neuroinflammation on hiPSC-derived CHOL neurons. Thus, we exposed hiPSC-derived CHOL neurons to Aβ fibril inflammation or to LRRK2-inhibited Aβ fibril inflammation for 3 days (Figure 5a). We first assessed the impact of LRRK2-mediated neuroinflammation on the rearrangement of neuronal shapes by analyzing the dendritic length and soma area of hiPSC-derived cholinergic (CHOL) neurons. At the end of differentiation, CHOL neurons were specifically identified and measured through ChAT immunolabeling. As shown in Figure 5, Aβ fibril inflammation leads to a significant reduction in the length of primary dendrites and the soma area of ChAT-positive CHOL neurons, the effects of which were not induced when glial cells were activated in the presence of LRRK2 MLi2 or PF inhibitor (Figure 5b,c). These findings indicate that Aβ fibril-mediated neuroinflammation contributes to neuronal shape modification and that glial LRRK2 participates in inducing these neuronal effects. Successively, we analyzed whether LRRK2-mediated neuroinflammation mediated cell degeneration even in CHOL neurons. In this regard, we found that Aβ fibril inflammation induced neuronal death, the effect of which was not observed in neurons exposed to LRRK2-inhibited inflammation (Figure 5d). Altogether, these results confirm the RNA-Seq data and suggest that, even under pathological conditions related to AD, LRRK2-mediated neuroinflammation impacts CHOL neuronal functions and vitality.

## 4. Discussion

The *LRRK2* gene is clearly associated with sporadic and familial late-onset PD. On the other hand, after the discovery of LRRK2 as a crucial regulator of neuroinflammation, growing evidence has started to link LRRK2 to other NDs with an inflammatory component. In this regard, it is well established that chronic neuroinflammation is a prominent feature of NDs; however, whether and how LRRK2-mediated neuroinflammation contributes to neuronal dysfunctions and damage is still unknown. In this study, we explored the impact of LRRK2-mediated neuroinflammation on neurons in a PD- and AD-related condition by using hiPSC-derived neurons exposed to inflamed glial medium as a cellular model. Starting with a transcriptomic approach, we found that LRRK2-mediated α-syn pff inflammation induced primarily oxidative stress and cell death in hiPSC-derived DA neurons. Conversely, in a context related to AD, we observed that LRRK2-mediated Aβ fibril inflammation was mainly involved in the rearrangement of neuronal shapes and the cell apoptotic processes of hiPSC-CHOL neurons. Taken together, our results indicate that LRRK2-mediated neuroinflammation contributes to neuronal dysfunctions and degeneration in both of these NDs.

Neuroinflammation is a well-understood condition in both PD and AD [6,28,29]. Reactive gliosis, activated microglia, and elevated levels of pro-inflammatory cytokines and chemokines have been reported in the brains of pre-clinical models and patients with PD [30,31,32,33] and AD [34,35,36,37,38]. The excessive activation of neuroinflammatory processes can worsen neuronal damage and lead to progressive neurodegeneration observed in these NDs [1]. Overall, these observations suggest that the modulation of the neuroimmune response might attenuate its detrimental effects on neurons and thus prevent the progression of NDs. Of interest, in this scenario, LRRK2, which is a positive regulator of neuroinflammation, could actively contribute to neuronal dysfunctions and damage in ND. In this study, to understand how LRRK2-mediated neuroinflammation impacts neuronal networks in PD and AD, we set up a cellular model with hiPSC-derived DA or CHOL neurons exposed to α-syn pff- or Aβ_1–42_ fibril-inflamed glial medium for several days, and we created a neuronal RNA-Seq profile. Interestingly, the analysis of the cellular pathways activated in hiPSC-derived neurons revealed that the neuroinflammatory response induced neuronal dysfunctions and damage, and that glial LRRK2 participates in mediating these detrimental effects. Specifically, α-syn pff-related neuroinflammation predominantly mediates oxidative stress, apoptosis, and a cellular response to the detoxification, which cellular processes are attenuated when hiPSC-derived neurons are exposed to LRRK2-inhibited inflammation. We functionally confirmed these results by evaluating neuronal ROS levels and neuronal phenotype and death. We found that hiPSC-derived DA neurons exposed to α-syn pff inflammation exhibited increased ROS levels, a suffering phenotype with a reduction in cell area and dendrite length, and an increase in neuronal death, the effects of which were not induced by the inflamed medium derived from cells activated in the presence of LRRK2 MLi2 or PF inhibitor. Aβ fibril-related neuroinflammation mainly mediated cell shape rearrangements, including cell size and axon extension, and apoptosis, to which cellular processes were attenuated when hiPSC-derived neurons were exposed to LRRK2-inhibited inflammation. Even for the AD context, we validated the RNA-Seq results, and we found alterations in neuronal shapes with reduced dendrite lengths and soma areas and an increase in neuronal death. Remarkably, these effects were not induced when glial cells were activated in the presence of LRRK2 MLi2 or PF inhibitor. Altogether, these results indicate that LRRK2, as a positive regulator of neuroinflammation, might contribute to inducing neuronal dysfunctions and damage in PD and AD. Importantly, our findings suggest that LRRK2 kinase inhibition may attenuate the inflammatory response and its related detrimental effects under pathological conditions. Supporting this hypothesis, targeting LRRK2-related inflammation has proven beneficial when translated into preclinical models. In this regard, we recently reported that LRRK2 kinase inhibition with MLi2 and PF molecules attenuates neuroinflammation, reactive gliosis, activated microglia, and cytotoxicity in mice with intracerebral injection of Aβ_1–42_ fibrils or α-syn pffs [12]. Accordingly, it has been shown that the exacerbated neuroinflammation and neurodegeneration observed in G2019S-LRRK2 transgenic rats could be mitigated by administering LRRK2 PF inhibitor [39]. Taken together, these observations support the hypothesis that lowering neuroinflammation processes, through the inhibition of LRRK2 kinase activity, might prevent their detrimental effects on neurons and thus cause them to be neuroprotective.

## 5. Conclusions

In conclusion, our study sheds light on the impact of LRRK2-mediated neuroinflammation on neurons in a context related to PD and AD and, importantly, suggests that targeting LRRK2 activity could be protective and beneficial for brain disorders with an inflammatory component.

## Figures and Tables

**Table 1:** Primer sequence.

Gene	Primer Sequence (5′-3′)
NANOG	Forward: TGCAAGAACTCTCCAACATCCT Reverse: ATTGCTATTCTTCGGCCAGTT
OCT4	Forward: GACAGGGGGAGGGGAGGAGCTAGG Reverse: CTTCCCTCCAACCAGTTGCCCCAAAC
ASCL1	Forward: CATCTCCCCCAACTACTCCA Reverse: AACGCCACTGACAAGAAAGC
DLX2	Forward: GCACATGGGTTCCTACCAGT Reverse: AACGCCACTGACAAGAAAGC
FOXG1	Forward: TGTTGACTCAGAACTCGCTGG Reverse: CTGCTCTGCGAAGTCATTGAC
LHX8	Forward: CAAGCACAATTTGCTCAGGA Reverse: CTGCTCTGCGAAGTCATTGAC
NKX2	Forward: GACACCATGAGGAACAGC Reverse: ACAGGTACTTCTGTTGCTTG
ChAT	Forward: GAGTACTGGCTGAATGACATG Reverse: AGTACACCAGAGATGAGGCT
VACHT	Forward: TACCCTACGGAGAGCGAAGA Reverse: CTGTAGAGGCGAACATGACG
GAPDH	Forward: GTTGTGGATCTGACATGCCG Reverse: GGTGGAAGAATGGGAGTTGC
HPRT	Forward: GGTGAAAAGGACCTCTCGAAG Reverse: GCTTTTCCACTTTCGCTGATG
ACTIN-β	Forward: CCTCTATGCCAACACAGTGC Reverse: CCTGCTTGCTGATCCACATC

## Data Availability

RNA-seq data (raw reads and processed counts) have been deposited to GEO. Other data supporting the conclusions of this article are available in the ZENODO repository (10.5281/zenodo.17078416) from the corresponding author upon reasonable request.

## Supplementary Materials

The following supporting information can be downloaded at: https://www.mdpi.com/article/10.3390/biom15091322/s1, Figure S1: Glial cells exhibited inflammatory and oxidative stress in response to α-syn pffs or Aβ fibrils; Figure S2: LRRK2 kinase inhibition; Figure S3: Time-dependent ROS generation of hiPSC-derived neurons exposed to inflamed GM; Figure S4: α-Syn pffs-related RNA Seq data analysis; Figure S5: Aβ-related RNA Seq data analysis. Table S1: DEGs after α-syn pffs-mediated inflammation. Table S2: GSEA after α-syn pffs-mediated inflammation. Table S3: DEGs after Aβ-mediated inflammation. Table S4: GSEA after Aβ-mediated inflammation. Western Blot contains original images of Figure 1 and Figure 2.

## Abbreviations

NDs	Neurodegenerative diseases
AD	Alzheimer’s disease
PD	Parkinson’s disease
LRRK2	Leucine-Rich Repeat Kinase 2
α-syn pffs	a-synuclein pre-formed fibrils
Aβ_1–42_ fibrils	Amyloid beta fibrils
LPS	Lipopolysaccharides
EDTA	Ethylenediaminetetraacetic acid
hiPSCs	Human induced pluripotent stem cells
DA	Dopaminergic
CHOL	Cholinergic
RNA-Seq	RNA sequencing
pSer935-LRRK2	Phosphoserine 935 LRRK2
IL-1β	Interleukin-1 beta
ROS	Reactive Oxygen Species
PBS	Phosphate-buffered saline
BDNF	Brain-derived neurotrophic factor
GDNF	Glial-derived neurotrophic factor
IGF-1	Insulin-like growth factor 1
cAMP	Cyclic adenosine monophosphate
DAPT	*N*-[1-(3,5-Difluorophenyl)ethyl]-*N*-[2-(3,5-difluorophenyl)acetyl]-l-alanyl-l-Phenylalanyl glycine
ChAT	Choline acetyltransferase
VAChT	Vesicular acetylcholine transporter
TH	Tyrosine hydroxylase
NANOG	Nanog Homeobox
Oct-4	octamer-binding transcription factor 4
ASCL1	Achaete-scute homolog 1
DLX2	Distal-Less Homeobox 2
FOXG1	Forkhead box G1
LHX8	LIM Homeobox 8
NKX2	NK2 Homeobox 1
GAPDH	Glyceraldehyde-3-phosphate dehydrogenase
HPRT	Hypoxanthine Phosphoribosyltransferase 1
GM	Glial medium
