# Supplementary material for: LRRK2-Mediated Neuroinflammation-Induced Neuronal Dysfunctions in a Parkinson’s and Alzheimer’s Disease Cellular Model
Source: Biomolecules. 2025 Sep 16;15(9):1322. doi: 10.3390/biom15091322 (PMC12467174; doi:10.3390/biom15091322)
Supplement: Supplementary file 1 [file biomolecules-15-01322-s001.zip › biomolecules-3754139-supplementary.pdf]

Supplementary information for

LRRK2-mediated neuroinflammation induced neuronal dysfunctions in a Parkinson's and Alzheimer's disease cellular model

Veronica Mutti[1], Giulia Carini[1], Moira Marizzoni[2], Alice Filippini[3], Federica Bono[4], Chiara Fiorentini[4], Samantha Saleri[2], Floriana De Cillis [5], Annamaria Cattaneo[2,5], Massimo Gennarelli[1,3], Paolo Martini[1], Isabella Russo[1,3,CA]

[1] Unit of Biology and Genetics, Department of Molecular and Translational Medicine, University of Brescia, Brescia, Italy;

[2] Biological Psychiatry Unit, IRCCS Istituto Centro San Giovanni di Dio Fatebenefratelli, Brescia, Italy;

[3] Genetic Unit, IRCCS Istituto Centro San Giovanni di Dio Fatebenefratelli, Brescia, Italy

[4] Unit of Pharmacology, Department of Molecular and Translational Medicine, University of Brescia, Brescia, Italy

[5] Department of Pharmacological and Biomolecular Sciences, University of Milan, Italy

[CA] To whom correspondence should be addressed:
Isabella Russo PhD, Unit of Biology and Genetics, Department of Molecular and Translational Medicine, University of Brescia, Viale Europa 11, 25123, Brescia, Italy.
Tel. +390303717461; Fax +390303701157; e-mail: isabella.russo@unibs.it

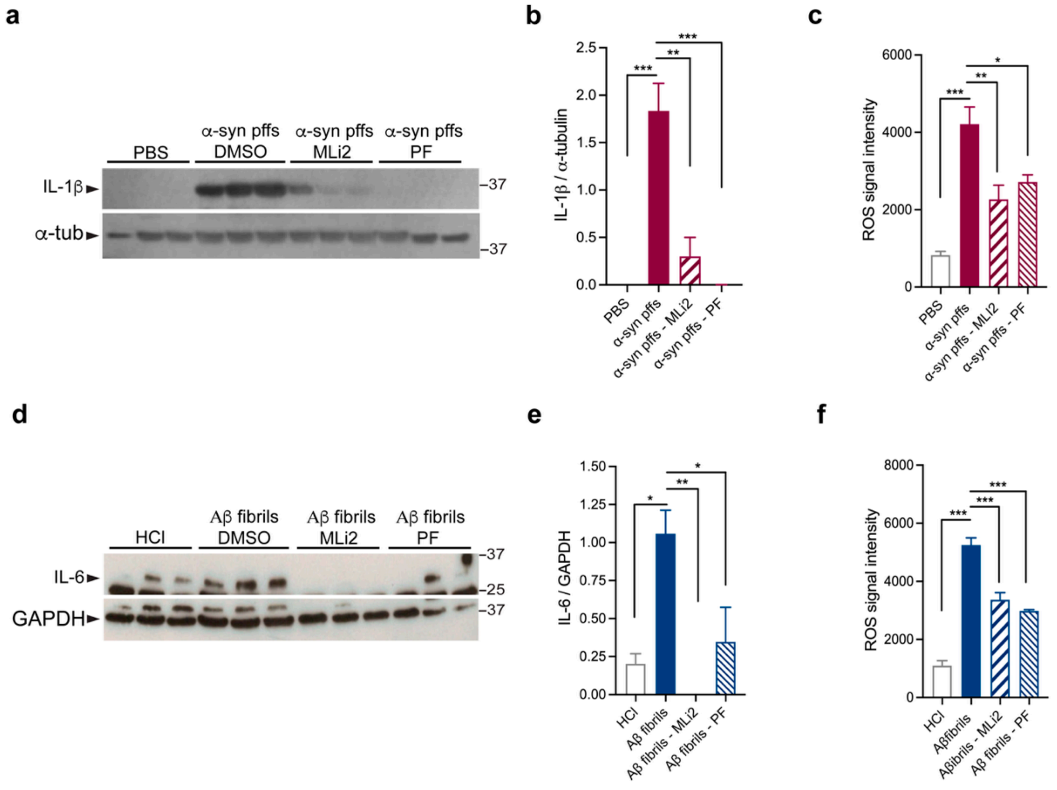

Suppl. Figure S1: Glial cells exhibited inflammatory and oxidative stress in response to α-syn pffs or Aβ fibrils. (a) Cell lysates of glial cells treated with α-syn pffs, α-syn pffs and MLi2, α-syn pffs and PF or PBS as control were subjected to immunoblotting using IL-1β and α-tub antibodies. **(b)** Quantification of IL-1β is normalized to α-tub protein. Data are representative of three independent experiments and are expressed as the mean ± SEM. Data were analyzed using one-way ANOVA followed by Tukey's post-hoc test, $**p<0.01$, $***p<0.001$. **(c)** Quantification of ROS generation in live glial cells treated with α-syn pffs, α-syn pffs and MLi2, α-syn pffs and PF or PBS as control. Data are representative of five independent experiments and are expressed as the mean ± SEM. Data were analyzed using one-way ANOVA followed by Tukey's post-hoc test, $*p<0.05$, $**p<0.01$, $***p<0.001$. **(d)** Cell lysates of glial cells treated with $A\beta_{1-42}$ fibrils, $A\beta_{1-42}$ fibrils and MLi2, $A\beta_{1-42}$ fibrils and PF or HCl as control were subjected to immunoblotting using IL-6 and GAPDH antibodies. **(e)** Quantification of IL-6 is normalized to GAPDH protein. Data are representative of three independent experiments and are expressed as the mean ± SEM. Data were analyzed using one-way ANOVA followed by Tukey's post-hoc test, $*p<0.05$, $**p<0.01$. **(f)** Quantification of ROS generation in live glial cells treated with $A\beta_{1-42}$ fibrils, $A\beta_{1-42}$ fibrils and MLi2, $A\beta_{1-42}$ fibrils and PF or HCl as control. Data are representative of five independent experiments and are expressed as the mean ± SEM. Data were analyzed using one-way ANOVA followed by Tukey's post-hoc test, $***p<0.001$.

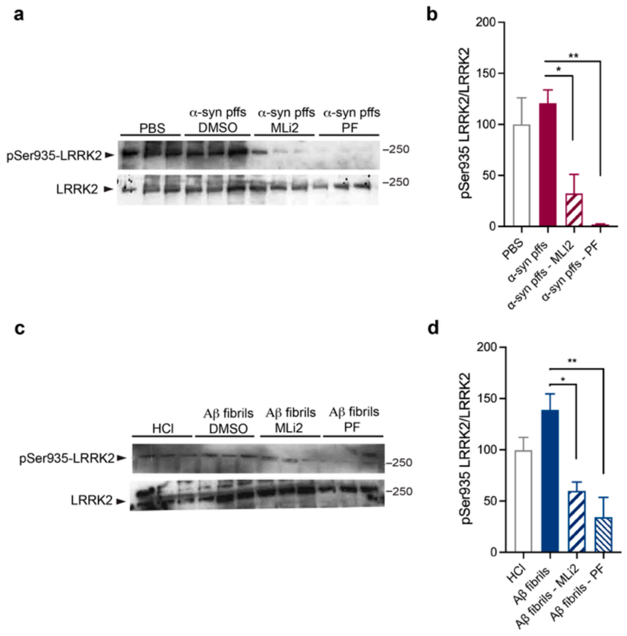

Suppl. Figure S2: LRRK2 kinase inhibition. **(a)** Cell lysates of glial cells treated with α-syn pffs, α-syn pffs and MLi2, α-syn pffs and PF or PBS as control were subjected to immunoblotting using pSer935-LRRK2 and LRRK2 antibodies. **(b)** Quantification of pSer935-LRRK2 is normalized to LRRK2 total protein. Data are representative of three independent experiments and are expressed as the mean ± SEM. Data were analyzed using one-way ANOVA followed by Tukey's post-hoc test, *p<0.05, **p<0.01. **(c)** Cell lysates of glial cells treated with Aβ fibrils, Aβ fibrils and MLi2, Aβ fibrils and PF or HCl as control were subjected to immunoblotting using pSer935-LRRK2 and LRRK2 antibodies. **(d)** Quantification of pSer935-LRRK2 is normalized to LRRK2 total protein. Data are representative of three independent experiments and are expressed as the mean ± SEM. Data were analyzed using one-way ANOVA followed by Tukey's post-hoc test, *p<0.05, **p<0.01.

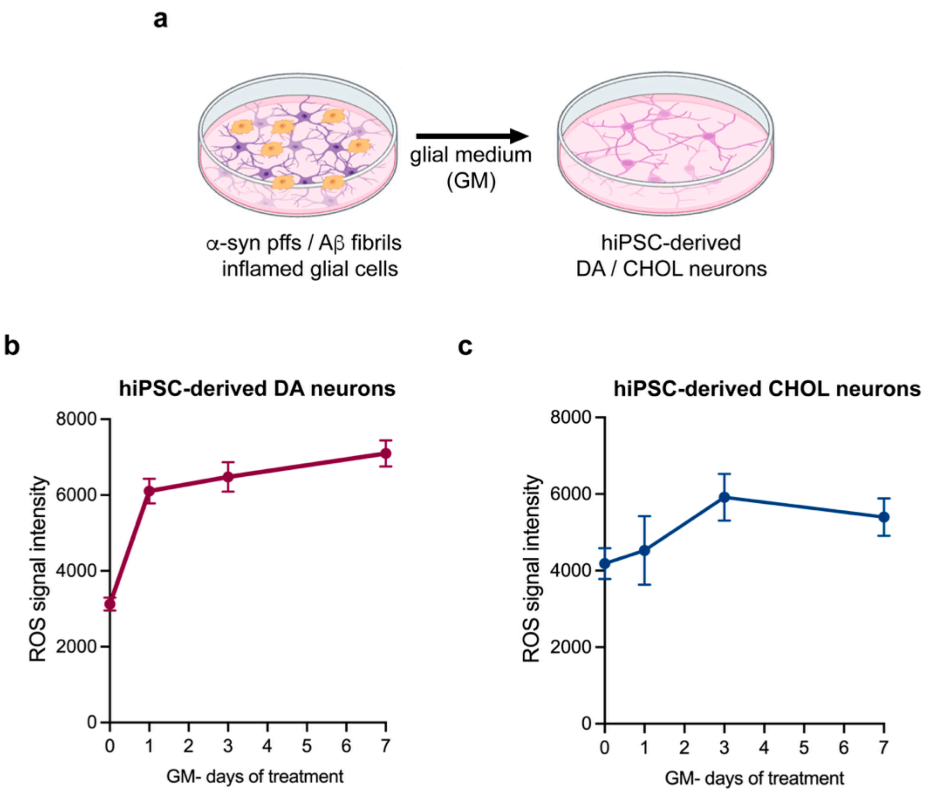

Suppl Figure S3: Time-dependent ROS generation of hiPSC-derived neurons exposed to inflamed GM. **(a)** Schematic representation of our cellular model. **(b)** Quantification of ROS generation in live hiPSC-derived DA neurons exposed to α-syn pffs inflamed glial medium for 0, 1, 3 and 7 days. Data are representative of four independent experiments and are expressed as the mean ± SEM. **(c)** Quantification of ROS generation in live hiPSC-derived CHOL neurons exposed to Aβ fibrils inflamed glial medium for 0, 1, 3 and 7 days. Data are representative of four independent experiments and are expressed as the mean ± SEM.

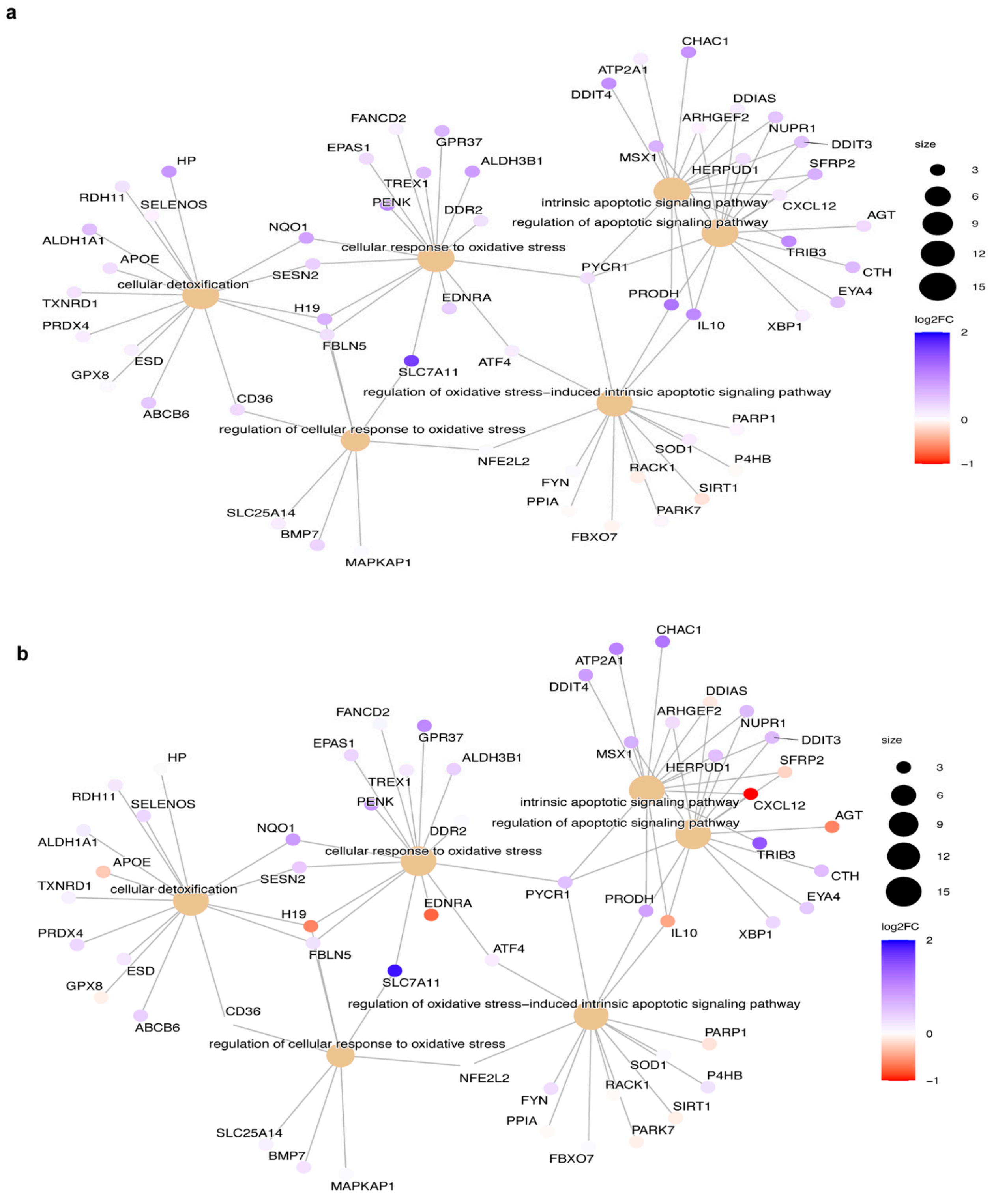

Suppl. Figure S4. α-Syn pffs-related RNA Seq data analysis. (a) Cnet-plot showing the top 15 genes with greater log2 fold change in the selected pathways for neurons exposed to α-syn pffs inflammation with LRRK2 MLi2 inhibitor. **(b)** Cnet-plot showing the top 15 genes with greater log2 fold change in the selected pathways for neurons exposed to α-syn pffs inflammation with LRRK2 PF inhibitor.

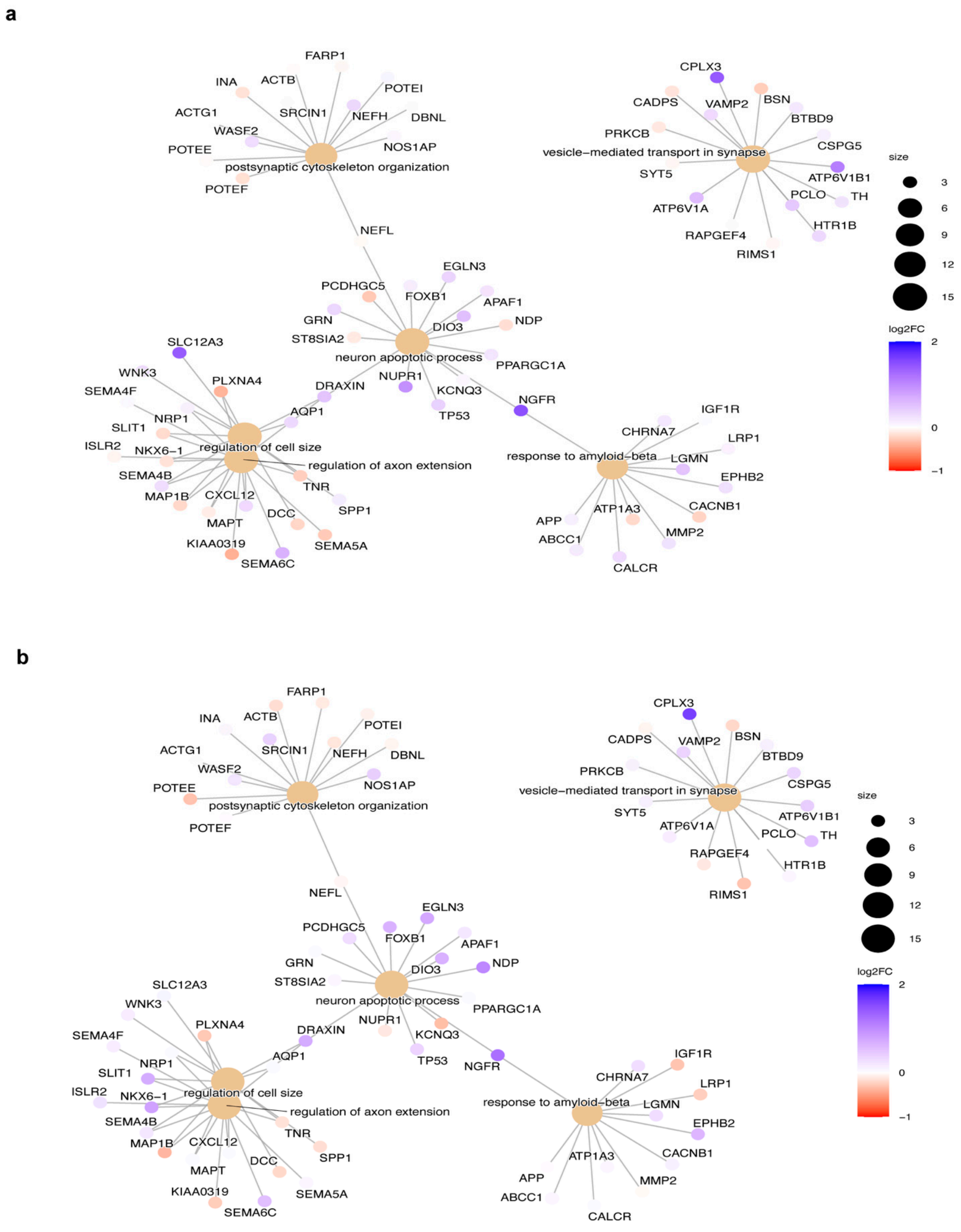

Suppl. Figure S5. Aβ -related RNA Seq data analysis. (a) Cnet-plot showing the top 15 genes with greater log2 fold change in the selected pathways for neurons exposed to Aβ inflammation with LRRK2 MLi2 inhibitor. **(b)** Cnet-plot showing the top 15 genes with greater log2 fold change in the selected pathways for neurons exposed to Aβ inflammation with LRRK2 PF inhibitor.
