# Supplementary material for: LRRK2-Mediated Neuroinflammation-Induced Neuronal Dysfunctions in a Parkinson’s and Alzheimer’s Disease Cellular Model
Source: Biomolecules. 2025 Sep 16;15(9):1322. doi: 10.3390/biom15091322 (PMC12467174; doi:10.3390/biom15091322)
Supplement: Supplementary file 1 [file biomolecules-15-01322-s001.zip › biomolecules-3754139-WB.pdf]

Western Blot Original Images

A

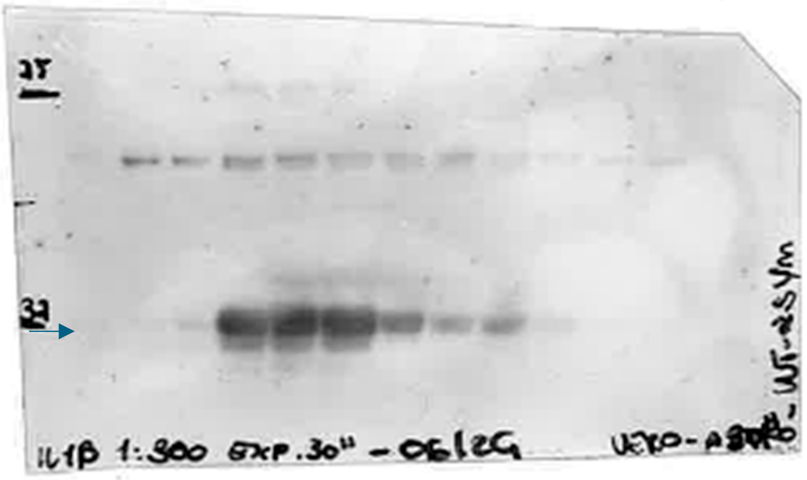

B

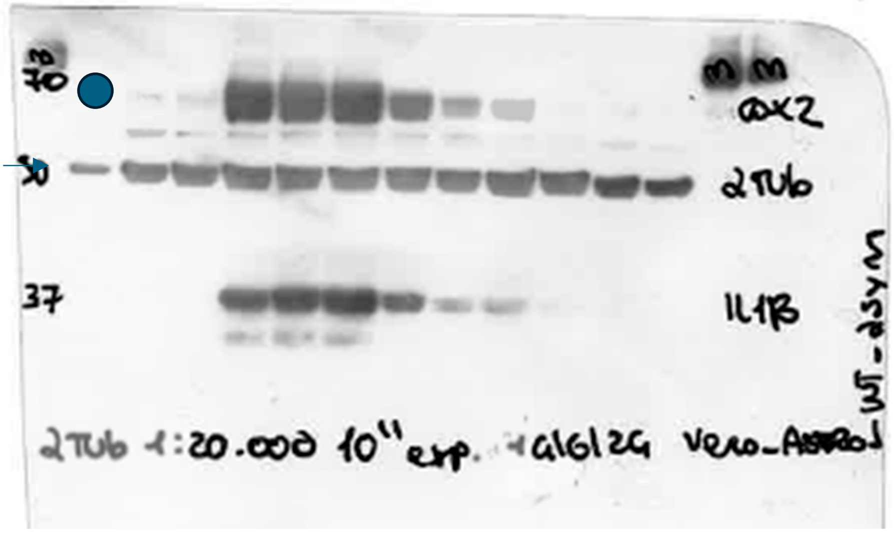

Additional Raw File 1 for Supplementary Figure 1a: Glial cell activation with α-synuclein pffs. Cell lysates of glial cells treated with PBS (lines 1-2-3); α-synuclein pffs (lines 4-5-6); α-synuclein pffs and MLi2 (lines 7-8-9); and α-synuclein pffs and PF (lines 10-11-12) were subjected to immunoblotting, detecting IL-1β (A, arrow) and α-tubulin (B, arrow). The upper band is the cyclooxygenase-2 marker, which we do not show in our manuscript (B, circle).

A

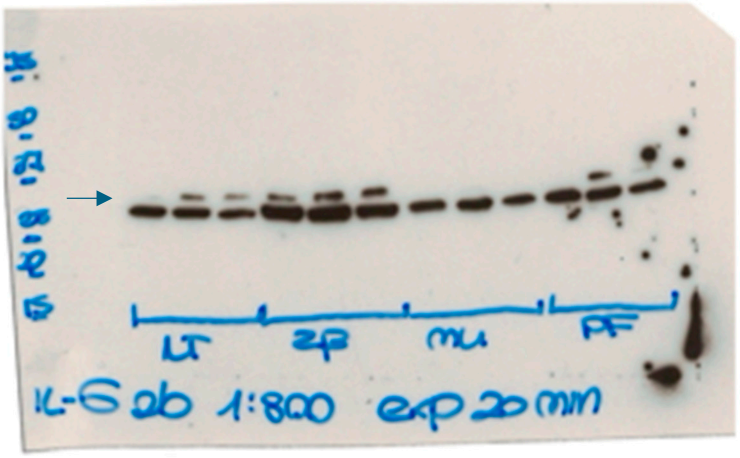

B

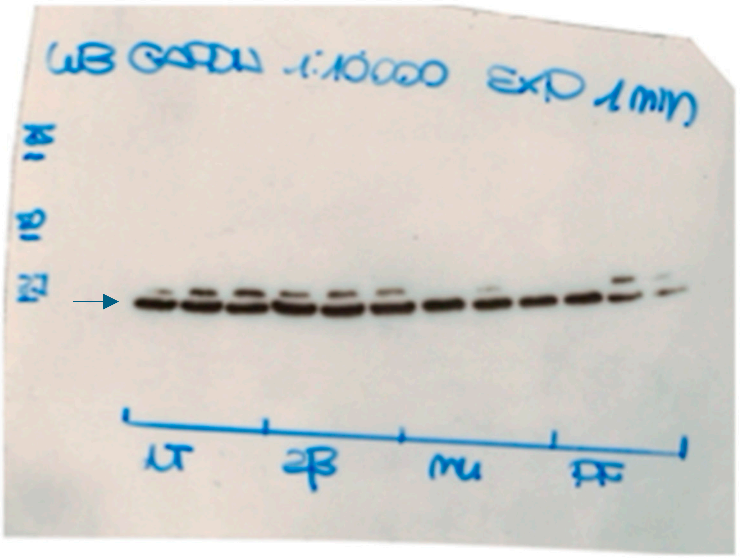

Additional Raw File 1 for Supplementary Figure 1d: Glial cell activation with $A\beta_{1-42}$ fibrils. Cell lysates of glial cells treated with HCl (lines 1-2-3); $A\beta_{1-42}$ fibrils (lines 4-5-6); $A\beta_{1-42}$ fibrils and MLi2 (lines 7-8-9); and $A\beta_{1-42}$ fibrils and PF (lines 10-11-12) were subjected to immunoblotting, detecting IL-6 (A, arrow) and GAPDH (B, arrow).

A

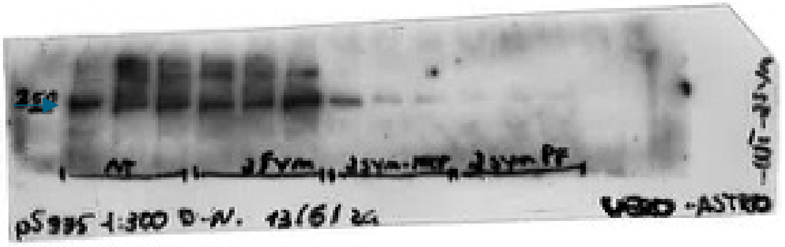

B

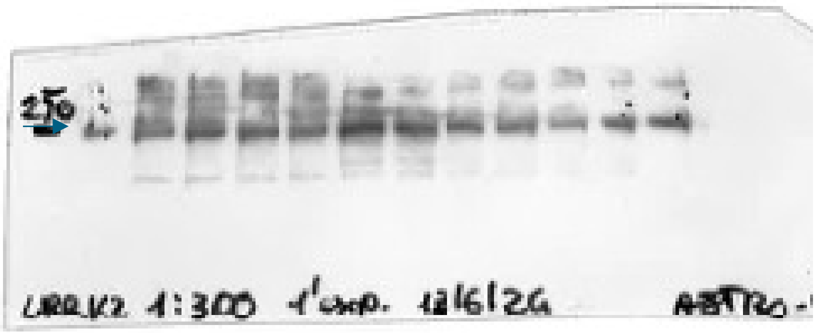

Additional Raw File 1 for Supplementary Figure 2a: Evaluation of Ser935-LRRK2 phosphorylation in glial cells treated with α-synuclein pffs. Cell lysates of glial cells treated with PBS (lines 1-2-3); α-synuclein pffs (lines 4-5-6); α-synuclein pffs and MLi2 (lines 7-8-9); and α-synuclein pffs and PF (lines 10-11-12) were subjected to immunoblotting, detecting pSer935-LRRK2 (A, arrow) and total LRRK2 (B, arrow).

A

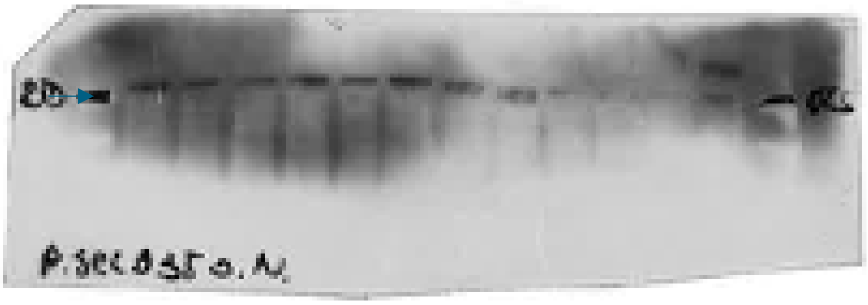

B

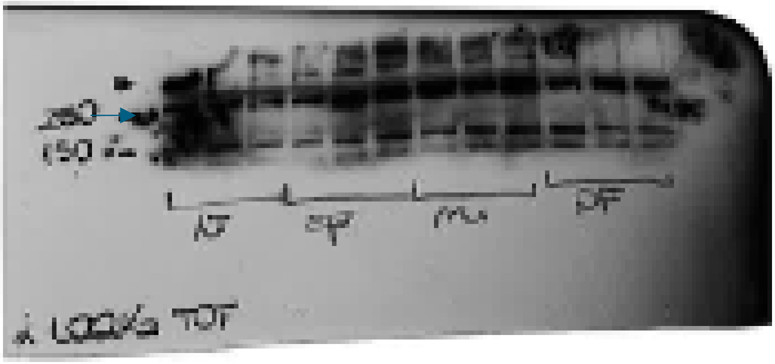

Additional Raw File 1 for Supplementary Figure 2c: Evaluation of Ser935-LRRK2 phosphorylation in glial cells treated with A$\beta_{1\text{-}42}$ fibrils. Cell lysates of glial cells treated with HCl (lines 1-2-3); A$\beta_{1\text{-}42}$ fibrils (lines 4-5-6); A$\beta_{1\text{-}42}$ fibrils and MLi2 (lines 7-8-9); and A$\beta_{1\text{-}42}$ fibrils and PF (lines 10-11-12) were subjected to immunoblotting, detecting pSer935-LRRK2 (A, arrow) and total LRRK2 (B, arrow).
